# Suicide loss of a close family member in childhood and suicide later in life—a nationwide study among Inuit youth in Kalaallit Nunaat

**DOI:** 10.1093/eurpub/ckag172

**Published:** 2026-09-21

**Authors:** Ivalu K Seidler, Janne S Tolstrup, Allison Crawford, Christina V L Larsen

**Affiliations:** National Institute of Public Health, University of Southern Denmark, Copenhagen, Denmark; National Institute of Public Health, University of Southern Denmark, Copenhagen, Denmark; Outreach and TeleMental Health, Centre for Addiction and Mental Health, Toronto, Canada; National Institute of Public Health, University of Southern Denmark, Copenhagen, Denmark

## Abstract

Suicide-related loss in close relations may lead to complicated grief and increase individual suicide risk. In Kalaallit Nunaat (Greenland), 60% of the population has lost a family member or a close friend to suicide. The study aimed to investigate the association between suicide-related loss of a close family member before the age of 10 and later suicide incidence. The study was a nationwide study using data from the central population register and register of causes of death including 25 663 individuals born between 1983 and 2012. Exposure was the loss of a mother, father, or one or more siblings before the age of 10 and outcome was suicide. Follow-up started at age 10 until the time of death, 40th birthday, or the study termination on 31 December 2022. Incidence rates and incidence rate ratios (IRR) were calculated using Poisson regression. Suicide-related loss of a mother was associated with the highest IRR of 3.4 (95% CI: 1.6–7.2). Suicide-related loss of a father was associated with an IRR of 1.9 (95% CI: 1–3.4), while suicide-related loss of one or more siblings was associated with an IRR of 2.4 (95% CI: 1.3–4.3). Transgenerational suicide transmission was identified in individuals who lost a close relative before the age of 10, with the highest IRR for loss of a mother. The high prevalence of suicide-related loss survivors stresses the need for public health strategies to ensure culturally relevant support.

## Introduction

Kalaallit Nunaat has one of the highest suicide rates globally [1]. These high rates are associated with a colonial history, rapid modernization, and societal disruption resulting in intergenerational trauma, inequity, and high prevalences of adverse childhood experiences, all of which are established risk factors for suicide and suicidal behavior [2–5].

### Suicide-related loss

The World Health Organization estimates that 700 000 people die by suicide every year [6]. Globally, suicide is the fourth leading cause of death among youth aged 15–29 years after road traffic incidents, tuberculosis, and interpersonal violence [6]. For every suicide, it is estimated that it will have a significant grief impact on at least six bereaved people, and more recent evidence suggests that that circle of impact is much larger, with each suicide-related loss significantly affecting 15–30 close family and friends, with additional impacts on up to 135 people [7, 8]. Suicide-related loss survivors often experience complicated grief, depression, post-traumatic stress disorder, and suicidal behaviors [9]. Complicated grief, also referred to in literature as traumatic or unresolved grief. A failure to resolve grief or loss, with symptoms that may include intense emotions, symptoms of depression, inability to function, difficulty accepting the loss, and in some cases traumatic symptoms such as flashbacks. Complicated grief is one mechanism that may partly explain the increased risk of suicide in those who experience bereavement from suicide [9].

Suicide-related losses that occur early in life can have impacts across the life course. Meta-analysis of the effects on children who have lost a parent to suicide demonstrates a two to three-fold increase in the likelihood of dying by suicide compared to children who have two living parents [10, 11]. Maternal suicide behavior is associated with a higher likelihood of suicide in offspring than paternal suicide behavior [10]. The age of exposure to parental suicide is an important factor for later suicide. A study on transgenerational transmission of suicide-related loss showed that suicide-related loss of a parent before the age of 14 years was associated with the highest cumulative hazard for suicide across different age groups for suicide-related loss when compared with parental suicide-related losses before age 1 and at ages 2–5, 6–14, and 15 years or older [12]. Differential impacts at various ages are potentially explained by the effect that such loss has on a child’s development at various stages. Critical periods in childhood can make neural development more sensitive to irreversible disruptions and alteration of neural circuits in the brain as a response to psychological and traumatic injury, impacting coping mechanisms and cognitive and emotional capabilities throughout the lifespan [13].

Many Indigenous Peoples regard the family as the center of life, and a close-knit family is often viewed as the premise for a healthy family [14]. This is also the case in Kalaallit society and culture, where people live closely together in smaller communities and where extended families, or families in which grandparents are the primary caregivers, are not uncommon [15]. The high prevalence of suicide in Kalaallit Nunaat thus affects the entire society, both through the impact on those exposed and due to the impact on the very fabric of society because of the ruptures of the family system. When communities experience clusters of youth suicides, the effect is of even greater magnitude [16].

In the Greenland Population Health Survey in 2018, 64% (*n* = 1198) reported that they had suffered a suicide-related loss by a close family member or a close friend (unpublished results). Results from the same survey showed that among youth who experienced a suicide-related loss of a near relation, 38% have had suicidal thoughts, and 30% have attempted suicide [17]. A report on grief in Kalaallit Nunaat concluded that complicated grief was prevalent among patients receiving therapy and that most patients viewed suicide-related loss as a part of the life that one had to endure [18]. The number of people affected by suicide in Kalaallit Nunaat and the potential rippling effects of suicide-related loss in close-knit communities and families create an imperative to investigate how suicide-related loss of a close family member during childhood affects the risk of youth suicide. The current study is the first nationwide study to investigate transgenerational transmission by examining suicide bereavement in childhood and later suicide incidence in a large Indigenous population.

### Aim

Based on nationwide register-data of Kalaallit Inuit, this study investigates whether suicide-related loss before the age of 10 years are associated with suicide incidence from childhood to young adulthood. We hypothesize that the suicide-related loss of a close family member will be associated with an increased risk of suicide prior to the age of 40 years, compared to both those who either experienced a non-suicide-related loss in childhood or no childhood losses.

## Methods

### Study design and data sources

The study is a retrospective nationwide register-based study on Kalaallit Inuit from 1983 until 2022. Kalaallit Inuit were identified as people born in Kalaallit Nunaat and living there at their 10th birthday. The study investigates suicide as an outcome among children, youth, and young adults aged 10–40 years since this is the age span where most suicides occur in Kalaallit Nunaat [16]. Suicide deaths in Kalaallit Nunaat have been registered since 1901 in various sources, while a nationwide registration was initiated in 1968 in the Register of Causes of Death [19, 20]. The register is updated regularly and validated against the Central Population Register (CPR). The register uses ICD-8 and since 1995 ICD-10 codes and has been evaluated as medium quality and covers all deaths [21]. All suicide deaths are verified by a physician, and the medical death certificates are then shared with the Danish Health Data Authority that code deaths to ICD codes and the information is recorded in the Greenland Register of Causes of Death. People are identified in the different registers based on their personal identification number (CPR number), allowing for register linkage. In the current study, information on birth, sex, death, family members, and migration was obtained from the CPR and linked with information on causes of death from the Register of Causes of Death.

### Research ethics

Register data is classified as personal data and was accessed through Statistics Greenland. Due to the legal protection of personal data, the data cannot be shared. The study is part of a PhD project approved by the Committee of Research Ethics in Greenland (KVUG 2018–16). All research has been done in accordance with the National Research Strategy of Greenland and the Ethical Research Guidelines from the University of Greenland. This research was led by an Indigenous first author who self identifies as Inuk and the research subject in question is an important and prioritized public health area in Kalaallit society. As a part of a PhD project all previous results of former studies have been widely disseminated to the public and communities, government, and other relevant stakeholders. The project and studies have integrated the knowledge and insights that has developed from the dissemination process and prior to that in the design phase of the project. Knowledge generated from the project has been integrated in the National Suicide Prevention Strategy. Authors acknowledge Indigenous Knowledge as equal to that of Western Science and combine the two in the manuscript.

### Study population

Children born in Kalaallit Nunaat from 1983 until 2012 were included in the study, and individuals who died before the age of 10 years or from causes other than suicide during the follow-up period were excluded.

### Exposure, outcome, and time

Suicide risk was investigated according to information on family and vital status. Exposure was defined as the suicide-related loss of a relative (mother, father, and sibling) or non-suicide-related loss among children aged 0–10 years. Non-suicide-related loss was included as a comparator based on a hypothesis that a suicide-related loss would have a greater impact on the suicide risk of the registrant than loss of a close family member due to other causes. The outcome of interest was suicide based on ICD-8 and 10 codes from the Register of Causes of Death (E950, E951, E953–E959 for ICD-8 and X60–X65, X70–X75, X78, X80, X83–X84 for ICD-10). In the study population, six people were missing information on their mother, and those six were categorized as missing in the loss of mother variable. In contrast, the 947 who did not have a registered father were categorized as *no father* in the loss of father variable. Each registrant born within the study period was followed from age 10 years until their death, their 40th birthday, or up to the study termination date on 31 December 2022. The age of 10 years was chosen as the start of follow-up since suicide before this age is a rare event [16]. The study design allowed for migration if vital status could be collected and verified at the end of the study period. Most of the migration from Kalaallit Nunaat is back-and-forth to Denmark, which has the same civil registration system as in Kalaallit Nunaat since both countries are part of the Kingdom of Denmark.

### Covariates

In Kalaallit Nunaat, young men aged 20–24 years are at the highest risk of suicide, with an average suicide rate of more than 400 per 100 000 people annually. The male-to-female ratio ranges between 1.5 and 4.3 between the ages of 15 and 39. However, recent research shows a narrowing of this gender gap with increasing female and decreasing male rates [16]. All four models were adjusted for sex due to this critical sex-related difference in rates. Sex was based on the sex assigned at birth as it is registered in the CPR. Since initial analyses showed no sex-related difference in the incidence rate ratios (IRR) for loss of a close family member no separate analyses were conducted. Based on the assumption that the loss of more than one family member would increase the risk of later suicide, the second model was further adjusted for the loss of other family members (mother, father, or sibling).

### Statistical analyses

All statistical analyses were performed using the statistical software Stata version 18 [22]. Suicide incidence was investigated by calculation of incidence rates and rate ratios using Poisson regression with age as time. No offset term was specified in the Poisson regressions.

## Results

In total, 25 663 individuals born between 1983 and 2012 were included in the study (Table 1), with a median age at the end of the study period of 27 years (range 10–40 years). In total, 774 individuals had full follow-up time from age 10–30 years, while 50% contributed with a follow-up time of 16 years (range from 0 to 30 years). During follow-up, 482 youth deaths by suicide were registered between the ages of 11 and 37 years, and the median age was 20 years.

**Table 1:** Characteristics at age 10 years among people born in Kalaallit Nunaat between 1983 and 2012.

*N*	25 663
**Sex, *n* (%)**	
Female	12 649 (49.3)
Male	13 014 (50.7)
**Birth year, median (min–max)**	1996 (1983–2012)
**Birth cohort, *n* (%)**	
1983–85	2572 (10)
1986–90	4875 (19)
1991–95	4986 (19.4)
1996–2000	4256 (16.6)
2001–05	3902 (15.2)
2006–10	3684 (14.4)
2011–12	1388 (5.4)

Before the age of 10 years, 0.4% of the children had lost a mother to suicide, while the corresponding proportion who lost a father to suicide was 1.1% (Table 2). Youth suicide rates varied from 115 suicides per 1 000 000 people annually among those who did not lose a parent and up to 431 per 100 000 among those who had lost a mother to suicide. Among those who had lost one or more siblings, 1.1% had lost them to suicide. Losing a close family member to suicide was associated with increased rate ratios of suicide in comparison to those who did not experience any loss, and the IRR was also significantly higher for suicide-related loss than non-suicide-related loss. The highest suicide IRR was seen among those who lost a mother, and suicide-related loss of a mother was associated with an IRR of 3.7 (95% CI: 1.8–7.8) in the model only adjusting for sex (model 1), which declined to 3.4 (95% CI: 1.6–7.2) when further adjusting for loss of other close family members (model 2). Suicide-related loss of one or more siblings was associated with a slightly higher IRR than suicide-related loss of a father (IRR = 2.4 vs. IRR = 1.9 in the fully adjusted model 2). The non-suicide-related loss of a close family member was only statistically associated with an increased IRR of 2.5 if the deceased was a mother compared to those who did not experience any loss.

**Table 2:** Vital status of close family members at age 10 years and later suicide rate and adjusted IRR after age 10 years in Kalaallit Nunaat from 1993 to 2022.^a^

	Vital status of family member	**Youth suicide** ^b^	Suicide rate per 100 000 people annually (95% CI)	**Model 1** ^c^ **IRR (95% CI)**	**Model 2** ^d^ **IRR (95% CI)**
**Parents**					
Alive	23 737	427	115 (104–126)	1 (ref.)	1 (ref.)
Dead (other causes)	584	17	183 (96–270)	1.6 (1–2.6)	1.5 (0.9–2.5)
Suicide	388	16	263 (134–391)	2.2 (1.3–3.6)	2.2 (1.3–3.6)
No father	947	21	133 (76–190)	1.1 (0.7–1.8)	1.2 (0.7–1.8)
**Mother**					
Alive	25 379	466	117 (106–128)	1 (ref.)	1 (ref.)
Dead (other causes)	172	8	318 (98–539)	2.6 (1.3–5.2)	2.5 (1.2–5)
Suicide	106	7	431 (112–750)	3.7 (1.8–7.8)	3.4 (1.6–7.2)
**Father**					
Alive	23 986	440	117 (106–128)	1 (ref.)	1 (ref.)
Dead (other causes)	436	10	140 (53–227)	1.2 (0.6–2.3)	1.1 (0.6–2.2)
Suicide	293	11	238 (98–379)	1.9 (1.1–3.5)	1.9 (1–3.4)
No father	947	21	133 (76–190)	1.1 (0.7–1.7)	1.1 (0.7–1.8)
**Sibling**					
Alive	25 090	464	118 (107–128)	1 (ref.)	1 (ref.)
Dead (other causes)	296	7	156 (41–272)	1.3 (0.6–2.8)	1.2 (0.6–2.6)
Suicide	277	11	299 (122–476)	2.5 (1.4–4.5)	2.4 (1.3–4.3)

a Differences in *n* for siblings and parents are explained by several siblings appearing with-in sets of parents.

b 1 missing for suicide in models including mothers.

c Model 1: adjusted for sex.

d Model 2: adjusted for sex and loss of other close family members.

## Discussion

This is the first nationwide study on the transgenerational transmission of suicide in an Indigenous population where suicide rates are often disproportionately high compared to non-Indigenous societies [23]. Among the 25 663 individuals in the study, 482 died by suicide before the age of 40 years, corresponding to almost 2%. The suicide rate was high among those who did not experience any loss and increased significantly in cases of suicide-related loss of a close family member [12]. The findings support our hypothesis that the suicide-related loss of a close family member in early life is linked to transgenerational transmission of suicide, with an increased incidence of suicide in adolescence and adulthood.

In our study, the most pronounced increase in the IRR was among those who had a suicide-related loss of a mother, where the IRR was between three to four times as high as those who did not experience loss of a mother. Suicide-related loss in one or more siblings increased the IRR by a factor of 2.5 compared to those who did not experience any such loss, while the suicide-related loss of a father doubled the rate ratio. Non-suicide-related loss of a close family member was only statistically associated with an increased IRR when the lost relative was a mother.

The findings of this nationwide study are in accordance with meta-analyses investigating the transgenerational transmission of suicide that found a two to three-fold increase in risk of suicide among offspring who had a suicide-related loss of a parent [10, 11]. In a Danish nationwide study, the suicide-related loss of a parent was associated with an IRR of 2.3 in the fully adjusted model; with a more pronounced increase in IRR found after adjusting for calendar period and current age group, with loss of father resulting in an IRR of 3, and loss of mother resulting in an IRR of 3.5 [12].

Future directions include understanding these impacts within cultural context, such as whether the difference in IRR of suicide-related loss of a father might be explained by the differences between the cultural roles of fathers partaking in the child’s upbringing in the two populations. The differences in the loss of a mother may also relate to the cultural importance of a maternal caregiver being stronger among Kalaallit Inuit. In addition, the Danish study was able to adjust for several potential mediators explaining some of the differences in estimates: familial psychopathology, socioeconomic factors, living with both parents, siblings, parents separated, and maternal and paternal age at the child’s birth [12]. Close cultural connection has been found as an essential protective factor for mental health and suicide among Indigenous peoples of the Circumpolar North [24, 25]. An integral part of Kalaallit community is *oqaaqqissaarineq*, the passing on of Kalaallit Inuit ways of knowing, lived experience and memories across generations that can help guide younger generations in life [26]. In many Indigenous societies, the mothers are often the conduits for passing on culture, and the loss of a mother would then imply the loss of a protective factor, potentially explaining the increased IRR among those who experienced a suicide-related loss of a mother in the current study. In many societies, including Kalaallit society, the mother is often the primary caregiver of the child, potentially explaining the higher IRR for the loss of a mother.

Due to this study being the first of its kind, comparison to other Arctic Indigenous peoples is not yet possible but will also be an important avenue for further research. Still, the loss of a close family member is a long-established risk factor for suicide with a potential genetic heritability of suicidal behavior [27, 28]. With elevated suicide rates in many Arctic Indigenous communities, the cumulative impacts of suicide-related loss cannot be underestimated. Suicide among youth is a particular concern, which may be exacerbated when suicide occurs in clusters among this age group [16]. High rates of suicide across the Arctic have been linked to colonization, societal, and cultural disruption, loss of identity, and intergenerational trauma, and suicides within families can contribute to intergenerational transmission of these impacts [2, 27–30]. Loss of cultural identity is of particular importance in Indigenous communities who experience high rates of youth suicides since culture function as an anker for youth when navigating life in a period of much development and vulnerability [26, 30, 31]. The close-knit society in Kalaallit Nunaat and the interdependence experienced in many communities, may exacerbate the impact of loss, felt and viewed as a collective loss. However, the collective loss and support from the community may fade away after the funeral, and many bereaved experience isolation, which may increase the risk of suicidal behavior [18].

The risks of suicide are multifaceted, with both biological aspects, such as genetic and epigenetic impacts of trauma, and psychological aspects, with a likely impact on early attachment [4]. Not only does the loss of a close family member cause a change in the social configuration of childhood and communities, but it also contributes to deepening a cultural *soul wound* [32]. Quantifying such complicated networks of risk and causes is not possible through the data available for the current study. Still, it is important to understand the intergenerational transmission of risk of suicide within a framework that considers the full cultural, historical, social, genetic, biological, and psychological range of risk and protective factors.

### Strengths and limitations

This study has strong methodological qualities due to the nationwide design enabling the calculation of suicide incidence and IRRs across all Inuit aged 10–40 years born in Kalaallit Nunaat between 1983 and 2012. With a population of only 56 000 individuals’ statistical power is a challenge in many analyses, especially with less prevalent events such as suicide. The use of national registers is, therefore, an essential tool to further elevate our knowledge of factors at play in significant public health and societal challenges, where suicide is one with devastatingly high impacts on the entire population. However, using registers for health research in Kalaallit Nunaat is part of a developing process, and the quality and availability of data need improvement. Significant covariates such as individual and family psychopathology, socioeconomic status, place of residence, and family dynamics were not available for this study, leaving a need for more research on their effects on suicide incidence. Information on suicide-related loss in close relations outside the family was not possible to obtain through registers despite being a known and important risk factor for suicide. Similarly, it was not possible to look at individual, family, and community protective factors within this study.

### Implications for future research 

The findings highlight the importance of understanding support systems in a population where approximately 60% reported experiencing suicide-related loss, a factor that has been associated with elevated suicide risk. Suicide-related loss was more common among individuals aged 35 years or younger and among those reporting severe social stress, suggesting that experiences of loss may be intertwined with broader social and life circumstances. Previous qualitative research has suggested that complicated grief may be prevalent in Kalaallit Nunaat [18]. Although the present study was not designed to examine grief processes directly, these findings indicate that grief and bereavement may be relevant considerations in understanding the consequences of suicide-related loss. The observed associations may reflect several overlapping mechanisms, including grief responses, accumulated adversity, social stress, and broader structural factors, all of which warrant further investigation. Strategies should incorporate the cultural aspects of grief, and prevention should also include known protective factors such as intergenerational connection, cultural knowledge and connection, and relationship with nature and land, while striving to ensure social equity [33, 34]. Evidence-based strategies to address suicide-loss are needed; emerging evidence from other populations suggests that with the right resources and intervention suicide-loss can also be transformed to a source of growth and strength, potentially interrupting these intergenerational impacts [35].

## Conclusion

Kalaallit Inuit experience transgenerational transmission of suicide when exposed to suicide-related loss before the age of 10 years, a transmission that is particularly present if the close relative lost is a mother. In general, the suicide incidence was high and increased by at least a factor of two or more when losing a close family member. In a society where the suicide rate is high and more than half of the population experiences suicide-related loss, there is an urgent need for public health strategies that provide support and tools to cope with loss and complicated grief in a culturally responsive manner. These findings contribute to further understanding the mechanisms underlying intergenerational transmission, particularly with reference to cultural differences.

## Data Availability

Data used for the study are the property of Statistics Greenland and can only be accessed by individual agreement. Key points Transgenerational transmission of suicide from parents to offspring is a serious problem increasing the risk of suicide two to almost four-fold for offspring. Suicide-related loss of a mother was associated with the highest increase in risk of suicide in offspring. Loss of a close family member causes a change in the social configuration of childhood and communities. The findings of the study identified a need to ensure support systems in a population where 60% experience suicide-related loss.
